# Reliability Estimation and Failure Analysis of LEDs with Various Currents and Correlated Color Temperatures for Identical Power Ratings: A Comparative Study

**DOI:** 10.1021/acsomega.5c00588

**Published:** 2025-02-17

**Authors:** J Lokesh, SG Kini, AN Padmasali, MG Mahesha

**Affiliations:** a Department of Electrical and Electronics Engineering, Manipal Academy of Higher Education, Manipal Institute of Technology, Manipal, Karnataka 576104, India; b Department of Physics, Manipal Academy of Higher Education, Manipal Institute of Technology, Manipal, Karnataka 576104, India

## Abstract

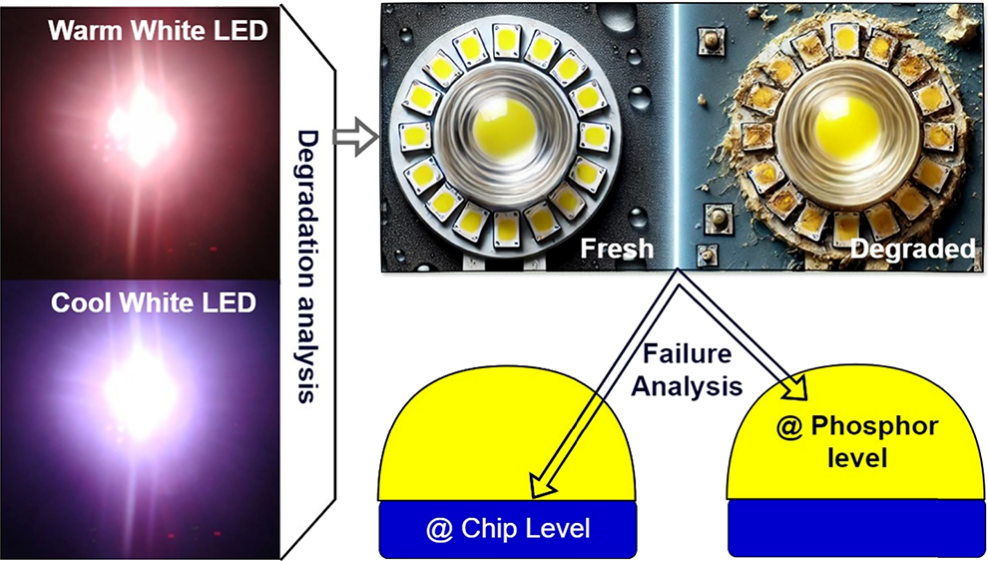

LEDs are available in a variety of correlated color temperatures (CCTs) to suit specific application needs. Manufacturers also offer LEDs with varying power ranges, and identical power ratings but different voltage and current specifications. While consumers prioritize power ratings and CCT, the lack of reliability information in manufacturer specifications makes LED selection challenging. A comprehensive lifetime performance and reliability analysis is crucial to address this gap, aiding consumers in selecting suitable LEDs for luminaire design and helping manufacturers identify failure causes through physics of failure analysis. This study evaluates the reliability analysis of 3.84 W phosphor converted high-power LEDs with various CCTs (warm white and cool white) and with varying voltage/current rating parameters for the same power through accelerated testing in an environmental chamber. The SPD, photoluminescence, capacitance–voltage (C–V), and current–voltage (I–V) measurements are used to conduct additional physics of failure analysis. The analysis showed that WW LEDs decay faster in lumen performance compared to CW LEDs and chip degradation is the primary cause for the reduced light output in both WW and CW LEDs. Among the different specifications for the same power rating in WW and CW LEDs, the lumen performance is observed to be almost similar. But there is comparable difference in Duv in CW LEDs.

## 1 Introduction

Light-emitting diodes (LEDs) are gaining popularity in a variety of applications, including data communication, automotive lighting, healthcare, and indoor and outdoor lighting, owing to their numerous advantages over traditional light sources, such as high luminous efficacy, freedom from IR and UV emission, long life, high-speed response, small volume, lower power consumption, and a wide range of controllability.

In general, in lighting applications, most LEDs have white emission. Presently, manufacturers commonly use a blue LED chip with yellow phosphor on top to produce white light owing to its simple construction, ease of control, and cost-effectiveness compared with methods using red, green, and blue LEDs. In practice, a wide range of correlated color temperature (CCT) LEDs can be produced by changing phosphor material properties such as thickness, concentration, and location. However, it is important to note that manufacturers produce LEDs not only with a wide range of CCTs but also with varying color rendering indices (CRIs), different power ranges, and the same power with different voltage and current ratings. Even though LEDs are available in a variety of configurations, users are more concerned about reliability, as this information is lacking in manufacturer information, which provides reliability data for only a limited number of LEDs. For these reasons, a reliability study is conducted on commercially available LEDs of the same power to assess their optical and electrical performance under varying CCTs and voltage/current ratings.

In general, L70 is a reliability criterion popularly used by manufacturers to define the lifetime of LEDs.^1,2^ Different studies have identified a variety of factors responsible for LED failure across different operating conditions.^3^ Reported that there is a reduction in the carrier injection efficiency due to the generation of some nonradiative recombination defects. Moreover, the behaviors of LEDs are quite different for higher temperature ranges (e.g., > 60 °C), and the degradation rate of the lumen values is much faster than that at room temperature.^4^ Explored the presence of cracks within the active region, the decline in the package optical properties, and the electrical characteristic variations. To develop complete knowledge for reliability assessment and failure studies, I–V characteristics, electroluminescence spectra, and thermal performance during high-stress conditions are recommended.^5^ Briefed that the change in the thermal resistance at lower temperatures is less variable than that at higher temperatures. The difference in thermal resistance is based on the package and chip structure. Additionally, the lateral structure LED has more heat generation than the flip-chip and vertical LEDs do, particularly at high temperatures, but there is no noticeable change at room temperature.^6^ Explains that the physics of failure-based prognostics and health management (PoF-based PHM). The high power LUXEON-K3 chip, which consists of GaN-based blue LEDs with multiple quantum well (MQW) structures, was used for the investigation. The study revealed that lumen degradation due to thermally induced and solder connection fatigue is the highest-priority and most critical mechanism in LEDs.^7^ Chemical processes are also responsible for many parametric or abrupt failures of solid-state lighting devices. The chemical processes include solder joint weakening and corrosion of wire bond pads, which can cause abrupt failure in LED devices, such as excessive loss of luminous flux, excessive chromaticity shifts or open circuits/short circuits.^8^ Notably, interface delamination and encapsulation (with or without phosphors) do not influence optical degradation during high-temperature aging. In contrast, the silver chloride produced during lead frame surface degradation plays a significant role in optical degradation, as more light is absorbed on the surface of lead frames.^9^ Physical failure analysis techniques are employed to understand the reasons for LED failure. Two failure mechanisms, X and Y, are identified. Mechanism X is due to mechanical destruction leading to a higher reverse saturation current, and mechanism Y is due to the dissolution of the phosphor and diffusion of the Zn activator.^10^ Reported the color shift mechanisms of an midpower white LED package under temperature, humidity, and electrical stresses. The current stress induces degradation in the LED die, which is analyzed on the basis of the reduction in peak intensity. Silicone carbonization due to temperature (85 °C) and humidity (85% RH) is the reason for the color shift in both the u’ and v’ directions.^11^ The failure modes and mechanisms of gallium nitride (GaN)-based cool white LEDs is presented in this study. The results revealed that (i) the reduction in the optical efficiency of LEDs is due to the generation of nonradiative combinations under constant current DC bias; (ii) the degradation in the optical parameters of GaN-based white LEDs results in reverse-bias stress or electrostatic discharge events; and (iii) high-temperature stress results in ohmic contact degradation and (iv) in PC (phosphor-converted) white LEDs, the optical property degradation implies the packaging/phosphors of LEDs.^12^ The study revealed that optical performance degradation is due to nonradiative recombination defects because the defect density is more sensitive to electrical stress than the light output and can be analyzed through a mathematical model.^13^ The high-power LED of 2.57W is used in this study. The results reported that silicone carbonization is due to the high local temperature generated by phosphor heating (due to the phosphor) rather than the use of only silicone. The temperature for carbonization of silicone is approximately 480 °C, which is far greater than the maximum junction temperature specified by the manufacturer in their datasheet.^14^ This study revealed that phosphor layer degradation contributes significantly to the overall decrease in lumen output (more than 60%) and that material degradation significantly alters the CRI and CCT.^15,16^ Studies used cool white LEDs. The results highlighted the changes in optical characteristics due to the thickness and concentration of the phosphor layer. As the phosphor layer thickness and particle concentration increase, the losses in optical degradation increase due to increases in the internal thermal resistance and temperature.^17^ A new moisture, electric, and temperature test is proposed and performed in this study. Two sets of high-power LEDs are used in this study. Compared with LEDs without a phosphor, white LEDs (with phosphor) show quick percentage degradation in the lumen, i.e., blue LEDs. The presence of moisture results in greater degradation in the lumen and a decrease in the forward voltage in terms of the V–I characteristics of the LEDs under operation than in the power-off conditions of the LEDs.^18^ This study explored the use of silicone-encapsulated epoxy microspheres to increase color temperature consistency in LEDs across various viewing angles. The results revealed that these microspheres did not reduce the reliability of the LEDs during high-temperature operation.^19^ The thermal degradation of an epoxy LED encapsulant due to external heat was examined in this study, revealing that higher phosphor concentrations accelerated the breakdown rates. The degradation rate of the epoxy encapsulant without phosphor was observed to be lower compared to that of the samples with phosphor mixed in the epoxy encapsulant.^20^ Phosphor-in-glass (PiG) encapsulants for high-power LEDs were evaluated for reliability via various phosphors in this study. The surfaces of PiGs with different phosphors exhibited differing levels of degradation, potentially due to structural incompatibility between the glass matrix and the phosphors.^21^ In this study, the conventional high-temperature solid-state approach was used to produce Ca_0.2_Sr_2.73_SiO_5_:0.07Eu^2+^ (CSSO) and Ba_0.2_Sr_2.73_SiO_5_:0.07Eu^2+^ (BSSO) phosphors. In adverse external conditions for warm LEDs, the BSSO phosphor demonstrated greater reliability and thermal stability than did CSSO.^22^ Ce:YAG ceramics were produced with varying concentrations of Gd^3+^ in this study. Increasing the Gd^3+^ concentration increased the reliability but not thermostability.^23^ For enhanced optical performance and reliability in white LEDs, phosphor-in-glass composites were explored. This innovative material offers the combined benefits of a glass matrix’s exceptional durability and the preserved luminescence properties of phosphors.^24^ Reported that there are important drifts in the I–V characteristics and confirmed that at a very early age, LED aging effects can be detected.^25^ Demonstrated that the degradation physics of LEDs at 70% RH, 85% RH, and 95% RH are completely dissimilar and that the use of 85% RH at 85 °C is inappropriate for extrapolation of the lifetime of LEDs.

A literature review revealed various mechanisms for the failure of LEDs in practice under different operating conditions, and various types of analysis have been performed. Studies have also demonstrated the contribution of phosphor in the overall failure of LEDs. The composition of phosphors in warm and cool white LEDs is different. The phosphor layer coating enables the transformation of blue light into specific color temperatures, categorizing them as warm white, cool white, or more, as desired. Since LEDs mainly consist of two key elements, analyzing their photoluminescence,^26,27^ capacitance–voltage (C–V),^28−30^ and current–voltage (I–V)^31−34^ characteristics is crucial for comprehending LED malfunctions. Photoluminescence analysis revealed the behavior of the phosphor component, whereas the C–V and I–V characteristics revealed the properties of the blue component. Merging these investigations yields a comprehensive understanding of LED failure, which involves both blue and phosphor components. LED manufacturers produce LEDs with different voltages and current configurations for the same power rating. As an example, a 1.96 W commercially available LED can be driven at 700 mA and 2.8 V and at 350 mA and 5.6 V.

Even though LEDs are available with various configurations, the selection of the right LEDs for luminaire design is an issue due to the lack of performance degradation analysis provided by LED manufacturers. In practice, LED manufacturers provide degradation analysis for a limited number of configurations; examples include (i) for only specific CCT LEDs and generalizations for other CCTs and (ii) despite the availability of LEDs in numerous configurations, only a single V & I configuration is utilized. Therefore, lifetime performance analysis of such LEDs is important, and this information is useful to LED manufacturers in selecting the right LEDs in the design of luminaires. For the above-mentioned reasons, a reliability study is carried out on commercially available LEDs of the same power to analyze their performance through optical and electrical characteristics: (i) with different CCTs and (ii) with different voltage and current configurations.

## 2 Methodology

**Figure 1:**
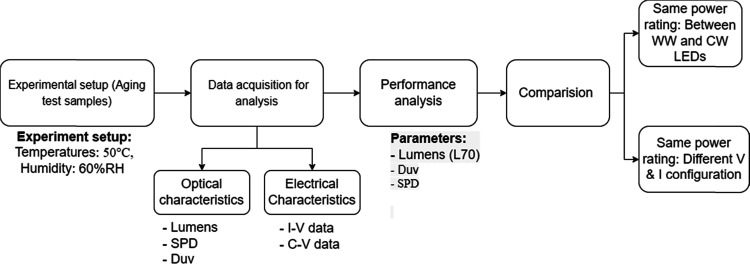
Methodology flowchart.

Reliability tests are performed on commercially available high-power white light LED packages to determine the optical deterioration and degradation mechanisms. The LED specifications are listed in Table 1, and the methodology used in this study is depicted in Figure 1. For this study, both warm and cool white LEDs of the same power rating with different voltages and currents are used. LED packages were soldered to metal-core printed circuit boards (MCPCBs). In this comparative analysis, the 6 V, 640 mA, and 24 V, 160 mA LEDs are referred to as HP1 and HP2, respectively. Warm white and cool white LEDs are indicated as WW and CW in the subsequent sections. Ten LED packages, HP1_WW, HP1_CW, HP2_WW and HP2_CW, were prepared to mitigate the effects of sample deviation during testing for the identified stress conditions. The experimentation and data acquisition details are presented in the subsequent sections.

**Table 1:** Specifications of the LEDs Considered for the Study

**power**	3.84 W	
**rated voltage**	6 V	24 V
**rated current**	640 mA	160 mA
**white shades**	warm white (3000K)	warm white (3000K)
	cool white (6500K)	cool white (6500K)

### 2.1 Experimental Setup and Data acquisition

**Figure 2:**
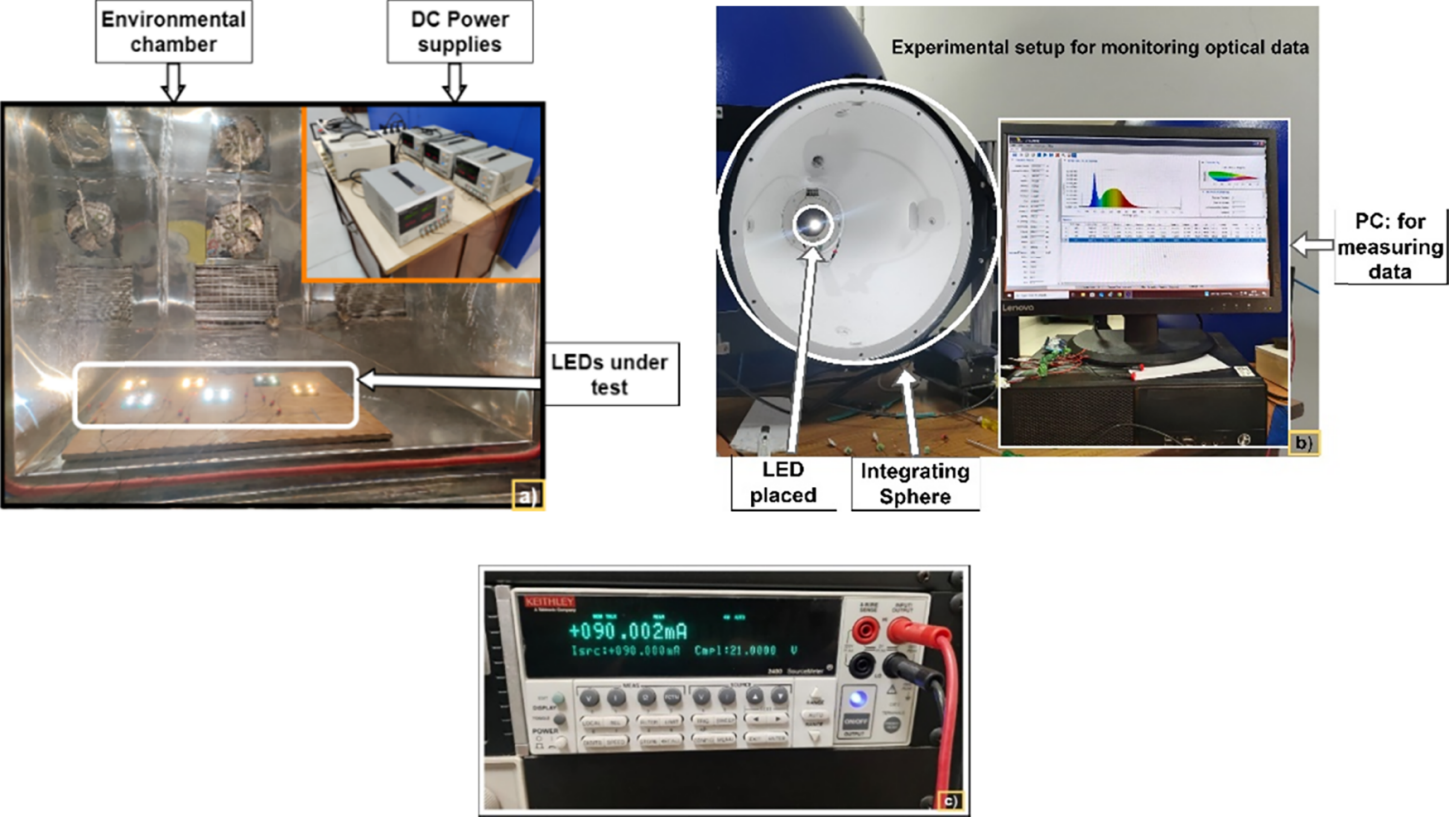
a) Experimental setup b) IllumiaPro LabSphere with a PC and c) Keithley 2400 source meter.

The experimental setup used in this study is depicted in Figure 2. The environmental chamber shown in Figure 2.a), is used to stress LEDs under controlled operating profiles. The optical data of the LEDs are measured using a 0.5 m diameter illumiaPro LabSphere as shown in Figure 2.b), which provides a wavelength accuracy of less than 0.3 nm, a resolution between 1 and 1.4 nm, and luminous flux measurements with 3% uncertainty. The electrical data (I–V) were measured with a Keithley 2400 source meter as shown in Figure 2.c). The source meter has V-ranges of 20 mV - 200 V, I-ranges of 10 nA - 1 A, and a basic accuracy of 0.012%. Similarly, a JASCO FP-8500 spectrofluorometer was used to determine the C–V characteristics.

The samples were aged in an environment chamber set to temperature and humidity stress levels of 50 °C and 60% RH, respectively. Before the LEDs are stressed at the selected temperature and humidity levels, the optical and electrical parameters are measured experimentally and are referred to as initial measurements (0 h). The test was stopped and the LEDs are allowed to cool down for 30 min prior to the data measurement. The optical and electrical characteristics of the aged samples are examined after the tests at random time intervals (hours) of 144, 224, 404, 504, 612, 738, 823, 1010, 1113, 1193, 1277, 1373, 1444, 1539, 1615, and 1705. The reliability test was stopped after 1705 h because all the LED categories chosen for the study’s lumen degradation met the L70 criteria. Individual parameter values are normalized to their initial values for all samples at various measurement points for performance analysis.

The optical parameters include the lumens, spectral power distribution (SPD), and Duv. Likewise, I–V and C–V data are included in the electrical parameters. Lumen performance is used to define the lifetime as per the reliability criterion of L70. Duv is a metric used to quantify the deviation of an LED’s color point from the blackbody locus on the CIE 1960 UCS (Uniform Color Space) chromaticity diagram. In addition, failure analysis of the LED chip and phosphor is performed individually. LED chip failure is analyzed on the basis of electrical characteristics. The series resistance (Rs) is calculated via I–V data and is used for analysis. The behavior of the phosphor layer was investigated through photoluminescence analysis via a JASCO FP-8500 spectrofluorometer. Photoluminescence is a widely used technique for evaluating the optoelectronic properties of semiconductors and other materials. Furthermore, the rate of spectral changes in phosphor emissions can be assessed by computing the centroid wavelength (λ̅) of the phosphor emission spectrum, as depicted by eq 1:^35^
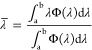
1

**Figure 3:**
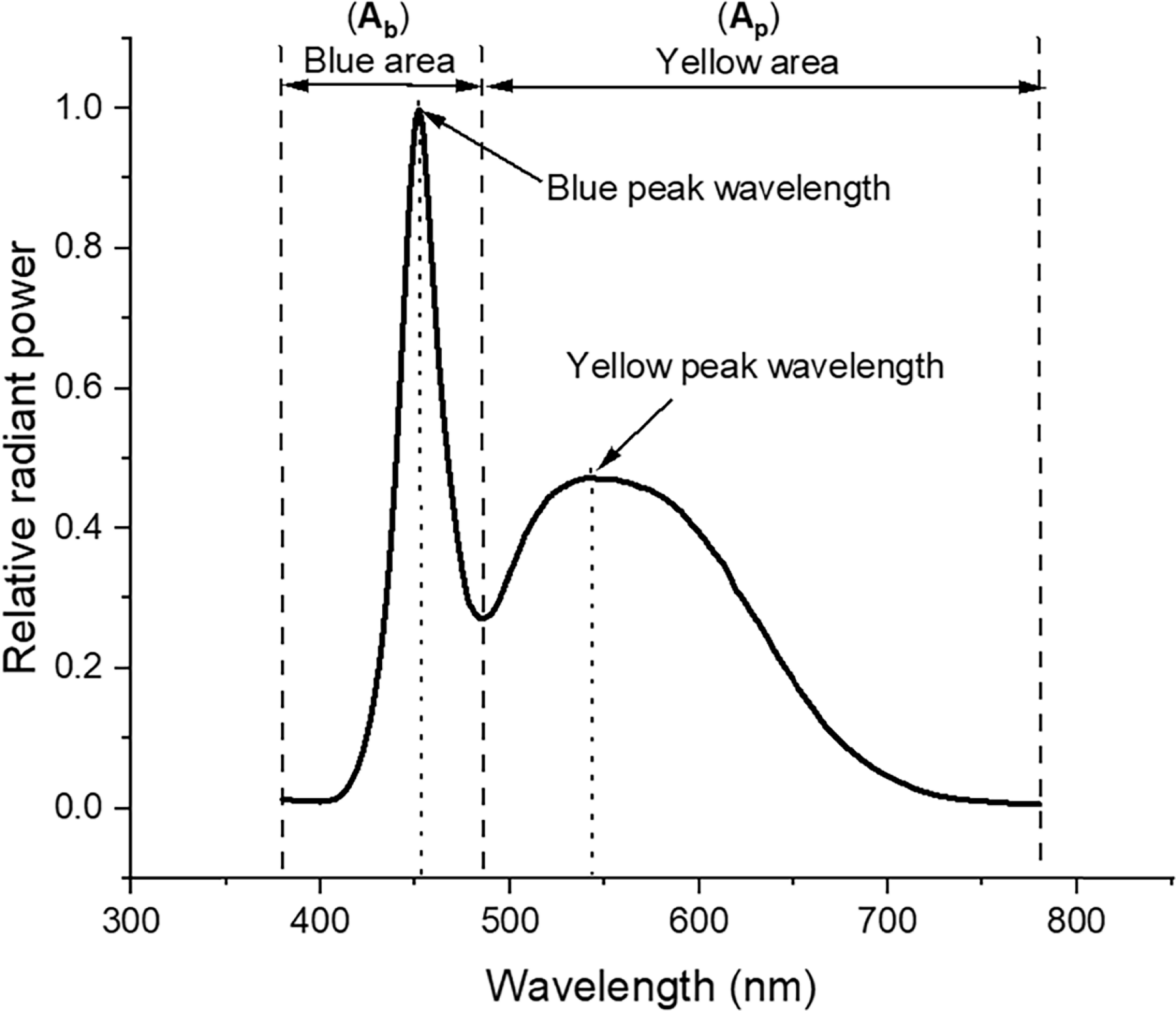
General SPD plot for cool white LED.

In eq 1, ‘a’ and ‘b’ represent the integration limits (470 and 780 nm for the phosphor peak), ‘λ’ denotes the wavelength, and ‘Φ(λ)’ represents the intensity at each wavelength. The results are also verified by extracting blue and phosphor-converted (down converted) spectral regions separately from SPD identified as blue area and yellow area as in Figure 3. This isolation helps in understanding the chip and phosphor degradation behavior of LEDs individually.

The overall comparative analysis is presented as a) optical degradation in terms of lumens, b) colorimetric analysis via Duv, c) separate blue and down converted light analysis via SPD, and d) chip degradation analysis via I–V and C–V characteristics and the extracted parameter Rs.

## 3 Results and Discussion

The reliability of the selected LEDs is assessed through optical degradation analysis via a lumen maintenance curve, with data measured up to the L70 criterion. The physics of failure analysis involves examining SPD, photoluminescence and characterizing I–V and C–V characteristics using the same duration of data collection.

### 3.1 Optical Degradation Analysis

**Figure 4:**
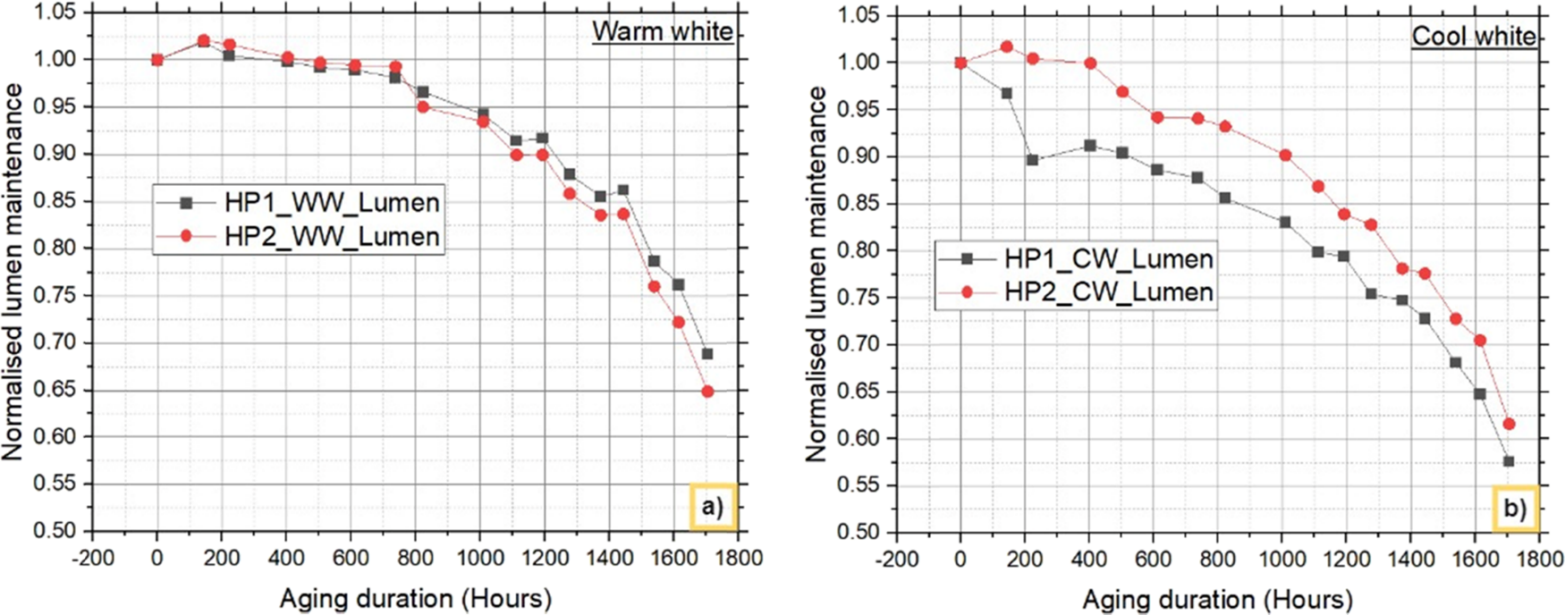
Lumen degradation: a) warm white LEDs and b) cool white LEDs.

For the analysis, the lumen output recorded at random intervals is utilized. Both HP1 and HP2 demonstrate exponential degradation kinetics in normalized lumens, as depicted in Figure 4. The findings indicate that the degradation rate of cool white light is significantly greater than that of warm white light, with a maximum difference of 7.314% observed at 1705 h.

The lumen degradation rates of the HP1_WW and HP2_WW LEDs after 1705 h are 31.152% and 35.102%, respectively. The absolute difference in the WW LED lumen performance is 3.955%. Similarly, at 1705 h, the percentages of the lumen of both the HP1_CW and HP2_CW LEDs were 42.421% and 38.401%, respectively. The absolute difference between cool white LEDs is 4.020%. According to these findings, the degradation rates of both cool white and warm white LEDs are comparable, with only a 0.065% difference. However, in WW LEDs, HP2_WW degrades faster than HP1_WW does, whereas in CW LEDs, HP1_CW degrades faster than HP2_CW does. Furthermore, the blue and yellow spectra are separated from SPD, and the degradation rates are compared to understand the reason for the difference in results and explained in the following section.

### 3.2 Spectral Analysis

**Figure 5:**
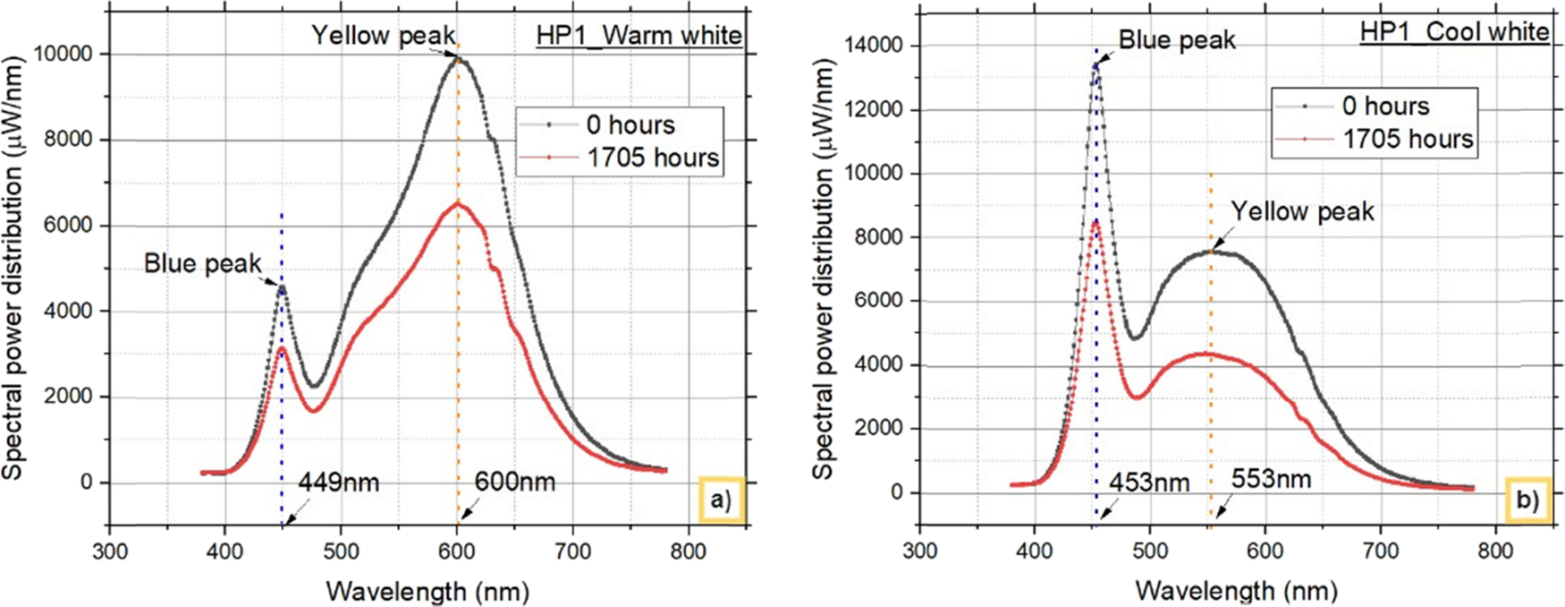
SPD from HP1 LEDs: a) warm white LEDs and b) cool white LEDs.

**Figure 6:**
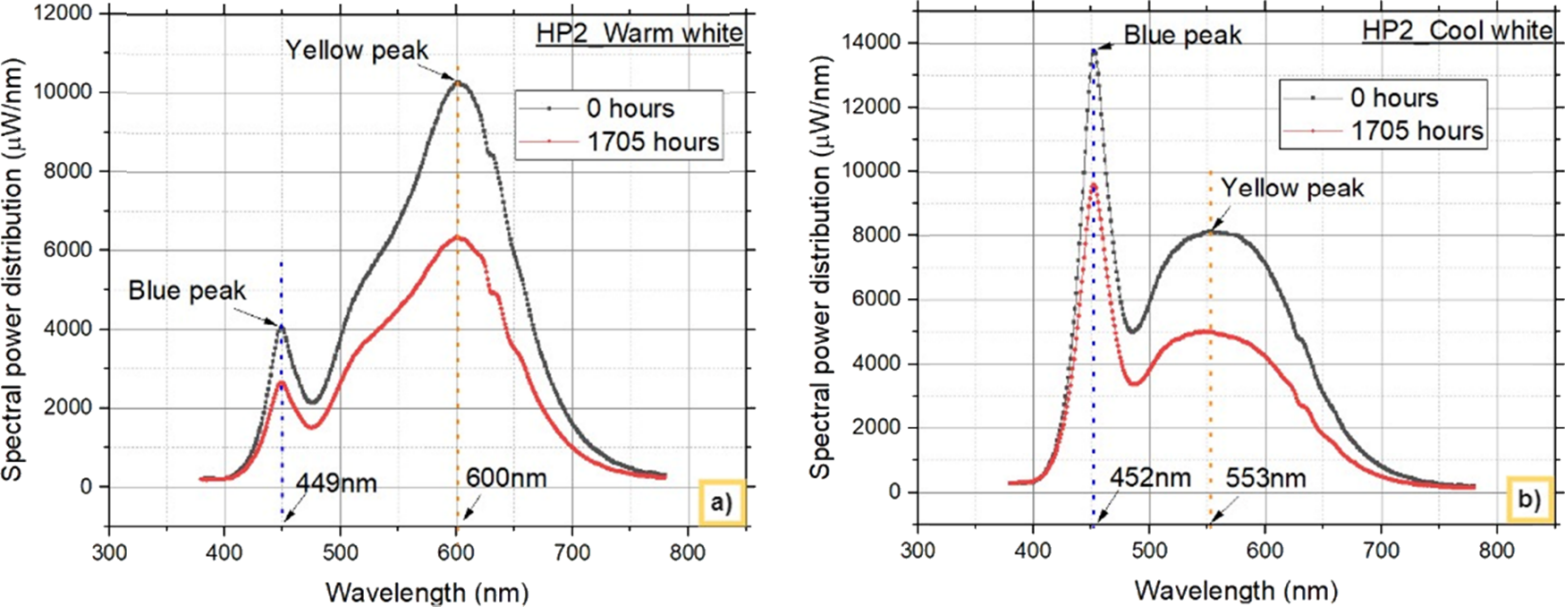
SPD from HP2 LEDs: a) warm white LEDs and b) cool white LEDs.

Figures 5 and 6 show the spectra of both warm white and cool white LEDs at 0 and 1705 h from both HP1 and HP2, respectively. The findings clearly show that the phosphor and chip materials in both CCT LED configurations do not cause a shift in the peak wavelengths emitted. However, SPDs indicate a decrease in lumen output over time, and the results are described in more detail in the previous section. Furthermore, to understand the reason for the difference in the lumen degradation rates between HP1 and HP2 LEDs with different CCTs, both blue light and downconverted light are separated from the SPD and analyzed.

**Figure 7:**
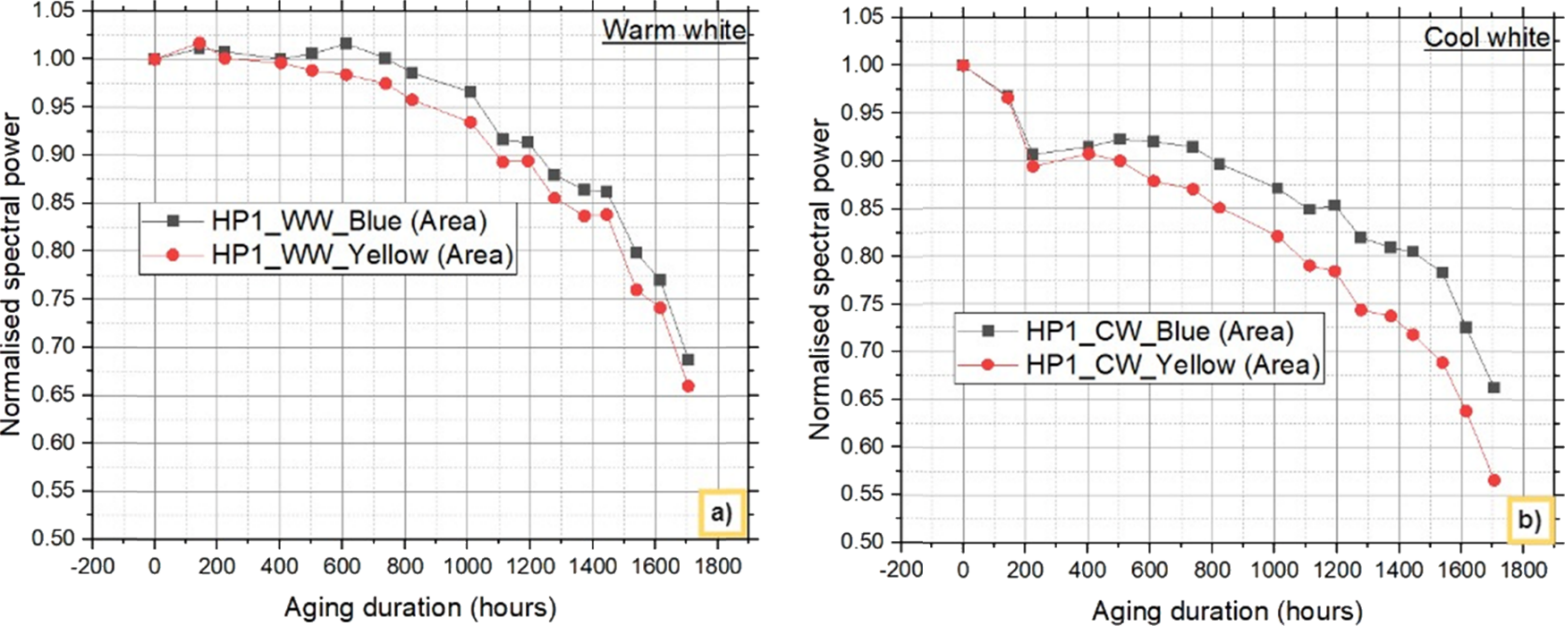
Blue and down converted light (yellow) from HP1 LEDs: a) warm white LEDs and b) cool white LEDs.

**Figure 8:**
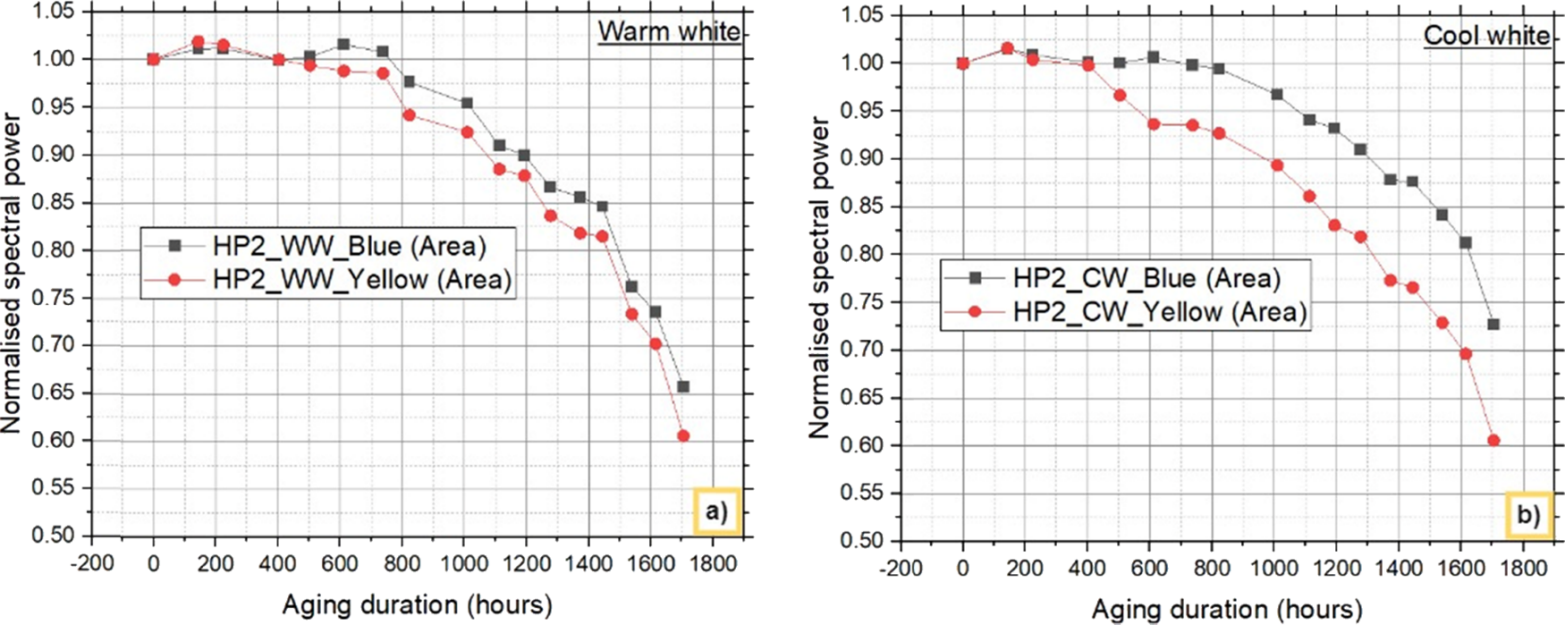
Blue and down converted light (yellow) from HP2 LEDs: a) warm white LEDs and b) cool white LEDs.

Figures 7 and 8 depict the degradation behavior of both blue light and down converted light emitted from warm white and cool white LEDs at 0 and 1705 h from HP1 and HP2, respectively. These findings indicate that, regardless of the LED configurations and different CCTs used, a reduction in blue light intensity is the primary cause of the observed decrease in lumens over time.

The contributions of the chip and phosphor to the decrease in the lumen over this period are further analyzed and shown. Tables 2 and 3 present the percentage decreases in blue and yellow phosphor, respectively, at 1705 h for the WW and CW LEDs. The difference in the % decrease in blue light between the HP1 and HP2 LEDs is attributed primarily to the difference in lumen degradation between the WW and CW LEDs. At 1705 h, the reduction in blue light from the HP1_WW and HP2_WW LEDs was 31.264% and 34.300%, respectively, whereas the reduction in yellow light was 2.8751% and 5.118%, respectively.

**Table 2:** Percent Degradation: HP1_WW & HP2_WW

	**HP1_WW**			**HP2_WW**		
**hours**	**lumen**	**blue**	**yellow**	**lumen**	**blue**	**yellow**
**0**	1	1	1	1	1	1
**1705**	0.688	0.687	0.659	0.648	0.657	0.605
**difference (0 and 1705 h) (%)**	31.152	31.264	34.015	35.107	34.300	39.418
**difference (blue and yellow) (%)**		2.751			5.118	

**Table 3:** Percent Degradation: HP1_CW & HP2_CW

	**HP1_CW**			**HP2_CW**		
**hours**	**lumen**	**blue**	**yellow**	**lumen**	**blue**	**yellow**
**0**	1	1	1	1	1	1
**1705**	0.576	0.662	0.566	0.616	0.727	0.606
**difference (0 and 1705 h) (%)**	42.421	33.804	43.439	38.401	27.300	39.418
**difference (blue and yellow) (%)**		9.635			12.118	

Similarly, at the same time point, the decreases in blue light from the HP1_CW and HP2_CW LEDs are 33.804% and 27.300%, respectively, with yellow light reductions of 9.635% and 12.118%, respectively. The results clearly show that the chip contributes more than the phosphor does to the failure of the LEDs chosen. To support these results, the electrical characteristics I–V and C–V are studied and briefly discussed in the next section. The results also indicate that the difference in yellow light is considerable and hence the variations in the colorimetric parameter, which is explained in a later section.

### 3.3 Physics of Failure Analysis

#### 3.3.1 C–V Measurement

**Figure 9:**
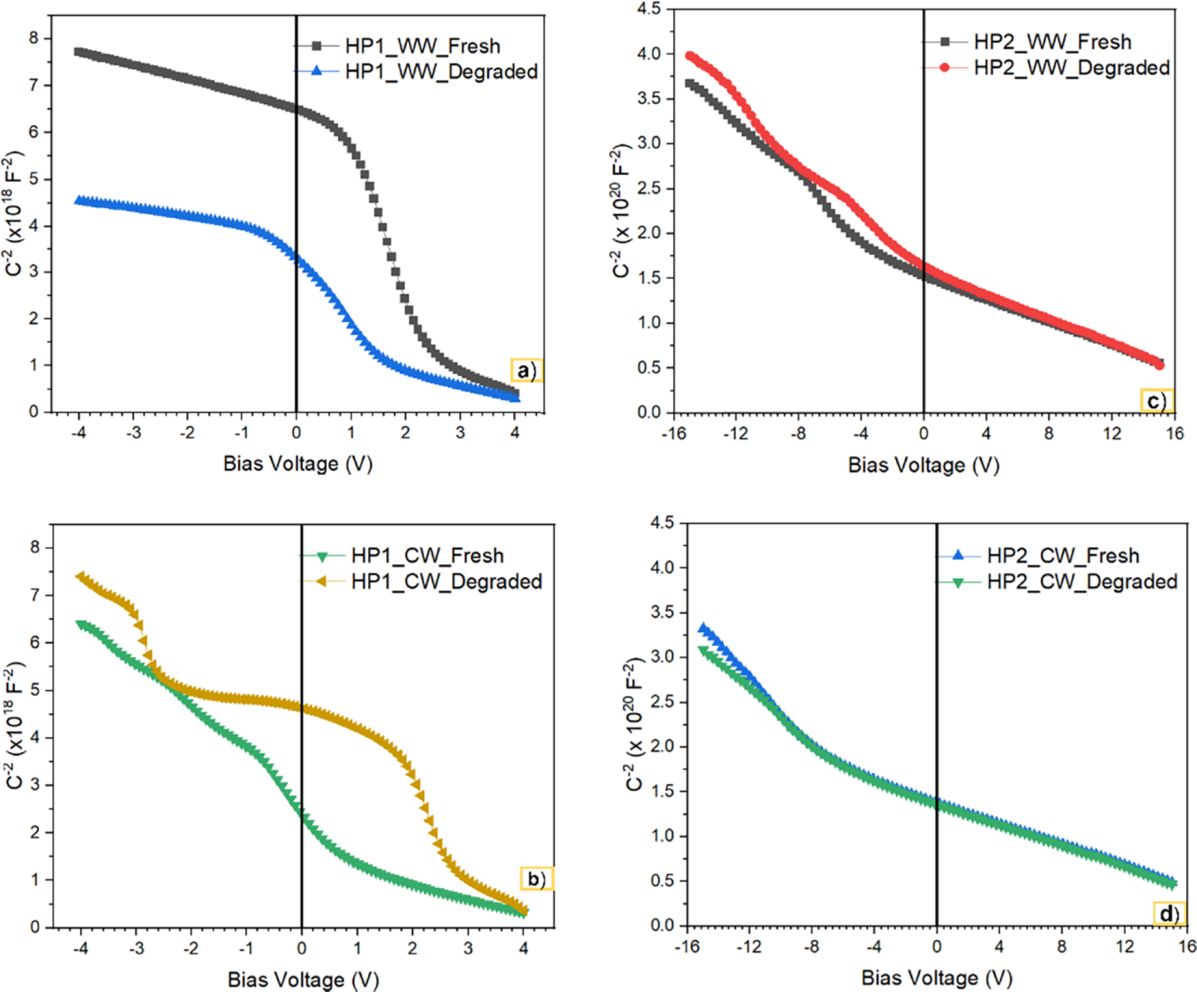
1/C^2^ vs V plot: (a) HP1 (6 V)_WW, (b) HP1 (6 V)_CW, (c) HP2 (24 V)_WW, and (d) HP2 (24 V)_CW.

C–V measurement is an ideal technique for analyzing the behavior of the diode junction, which provides information about the density of charge carriers, defect centers, and built-in potential^28_30^. The depletion layer capacitance (C) is related to the applied bias voltage (V) according to eq 2.^28^
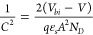
2where *V*_*bi*_ is the built-in potential, *q* is the electronic charge, ε_*s*_ is the permittivity, *A* is the diode area, and *N*_*D*_ is the carrier density. The 1/C^2^ vs V plot is shown in Figure 9. Compared with the zero-hour LED, the degraded 6 V warm white LED shows an increase in C–V behavior in the reverse bias region. The increase in the junction capacitance in the reverse bias region suggests the formation of more traps due to prolonged electrical stress and thus deterioration of the device’s performance.^36^ These traps can act like recombination centers or scattering sites for charge carriers. In the case of GaN-based LEDs, electrical stress may lead to the formation of nitrogen interstitials (N_i_), which can act as electron traps. Theoretical studies have investigated the formation of nitrogen interstitial (N_i_) defects in GaN.^37^ Due to its relatively low formation energy, the nitrogen interstitial (N_i_) is one of the most common defects in GaN, after prolonged exposure to the electrical stress. When lattice nitrogen is displaced into an interstitial position, it can form a Frenkel pair (N_i_–V_N_). These pairs are stable and do not annihilate easily, although annihilation begins to occur at 673 K. The formation of such interstitial defects leads to a reduction in the quantum efficiency of the device.

#### 3.3.2 I–V Characteristics

**Figure 10:**
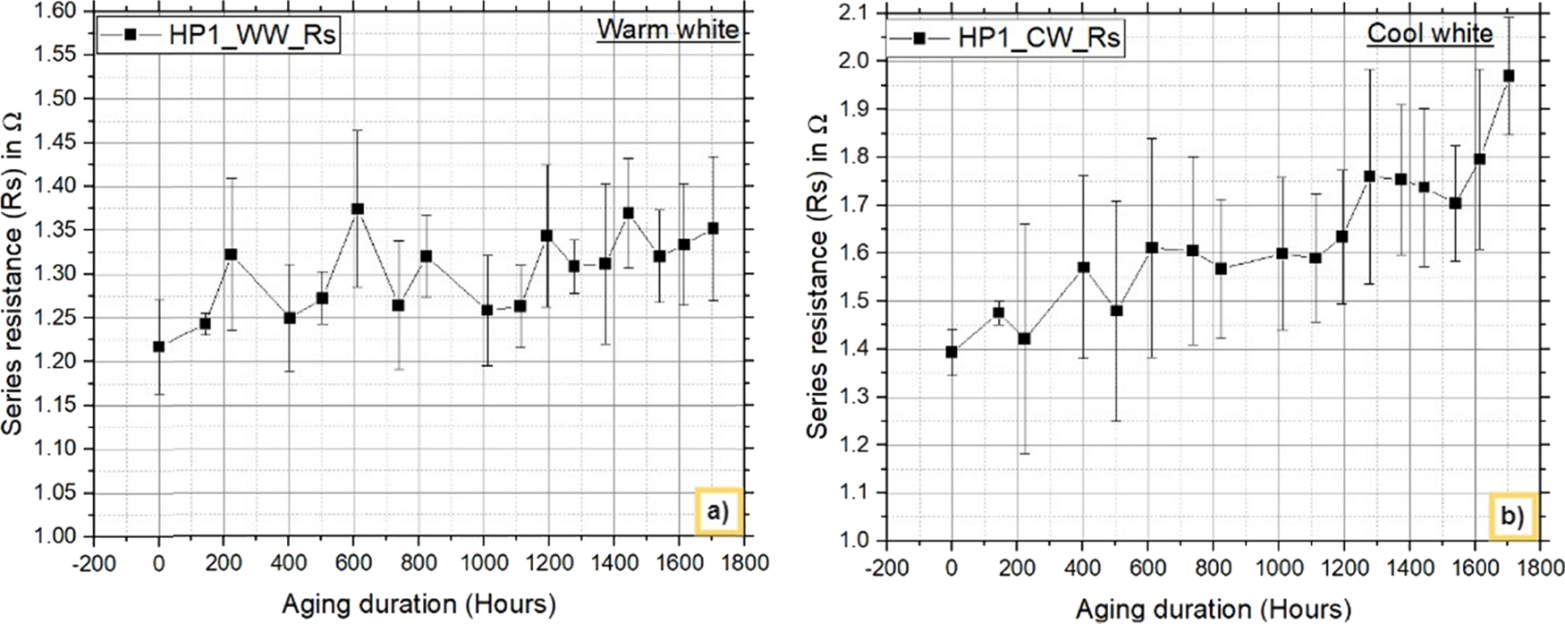
HP1: Variations in the series resistance (Rs) with aging for a) warm white and b) cool white LEDs.

**Figure 11:**
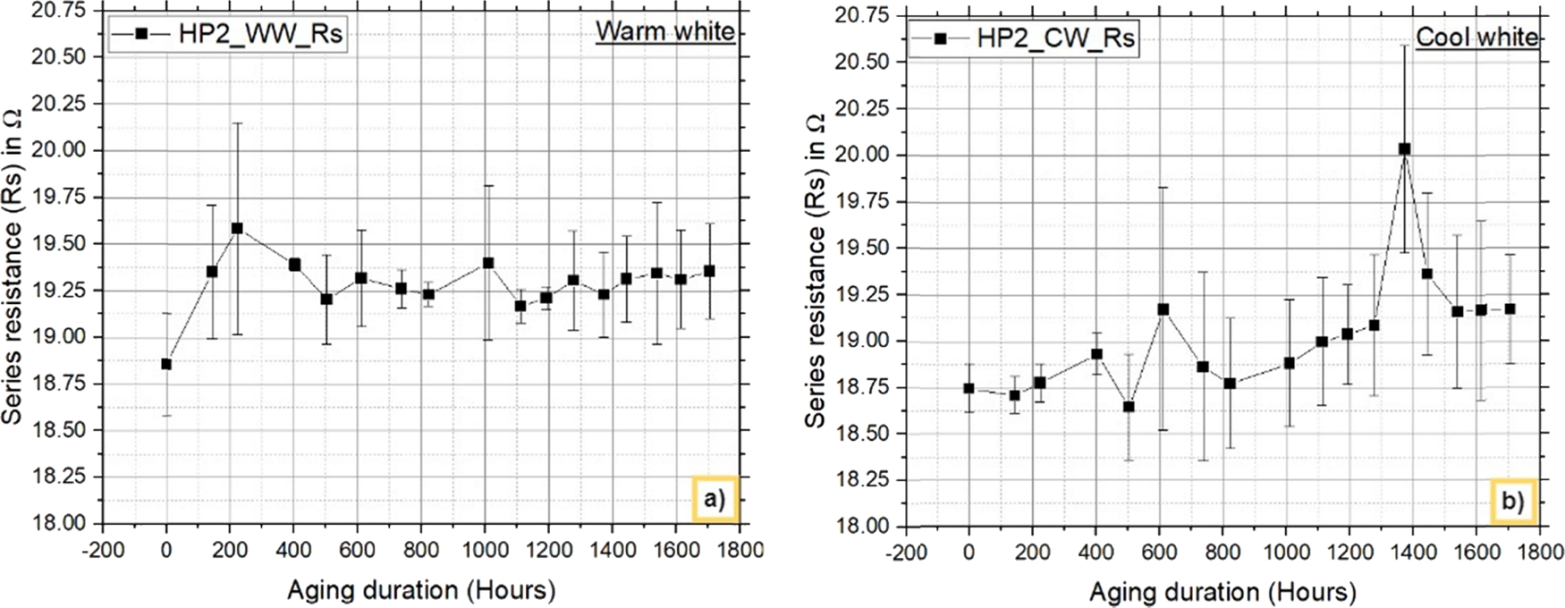
HP2: Variations in the series resistance (Rs) with aging for a) warm white LEDs and b) cool white LEDs.

The electrical (I–V) characteristics are measured at each readout time, and the device characteristic “Rs” is calculated. Figures 10 and 11 show the nature of “Rs” over the aging hours. The results clearly reveal an increase in Rs for both warm white and cool white LEDs and also for both HP1 and HP2 LEDs. The maximum standard deviation was found to be less than 10%. The increased series resistance impacts the emission efficiency of the blue chips, leading to lumen degradation in the LED packages.^38^ The increase in series resistance is caused by thermally activated interdiffusion between metal–metal and metal–semiconductor interfaces.^39,40^

The variations in Rs are compared and are given in Table 4. The results indicate that the increase in the Rs value is greater for HP2_WW than for HP1_WW after 1705 h. The difference in the Rs value between HP1_WW and HP2_WW is 0.364 ohms. Similarly, in the CW LED comparison, the increase in the Rs value is greater with HP1_CW than with HP2_CW. The difference in the Rs value between HP1_CW and HP2_CW is 0.1563 ohms. The smaller difference in the increase in the Rs value between the CW LEDs and WW LEDs resulted in considerable degradation under down converted light, and the results revealed a difference in lumen degradation between HP1_CW and HP2_CW. The contribution of phosphor results in different results between WW and CW LEDs, which is verified considering Duv and explained in the following section.

**Table 4:** Series Resistance (Rs) in Ω: HP1_WW & HP2_WW

	**warm white**		**cool white**	
**hours**	**HP1_WW**	**HP2_WW**	**HP1_CW**	**HP2_CW**
**0**	1.217	18.858	1.393	18.747
**1705**	1.352	19.357	1.969	19.181
**difference**	–0.135	–0.499	–0.576	–0.423

### 3.4 Colorimetric Analysis-Duv

**Figure 12:**
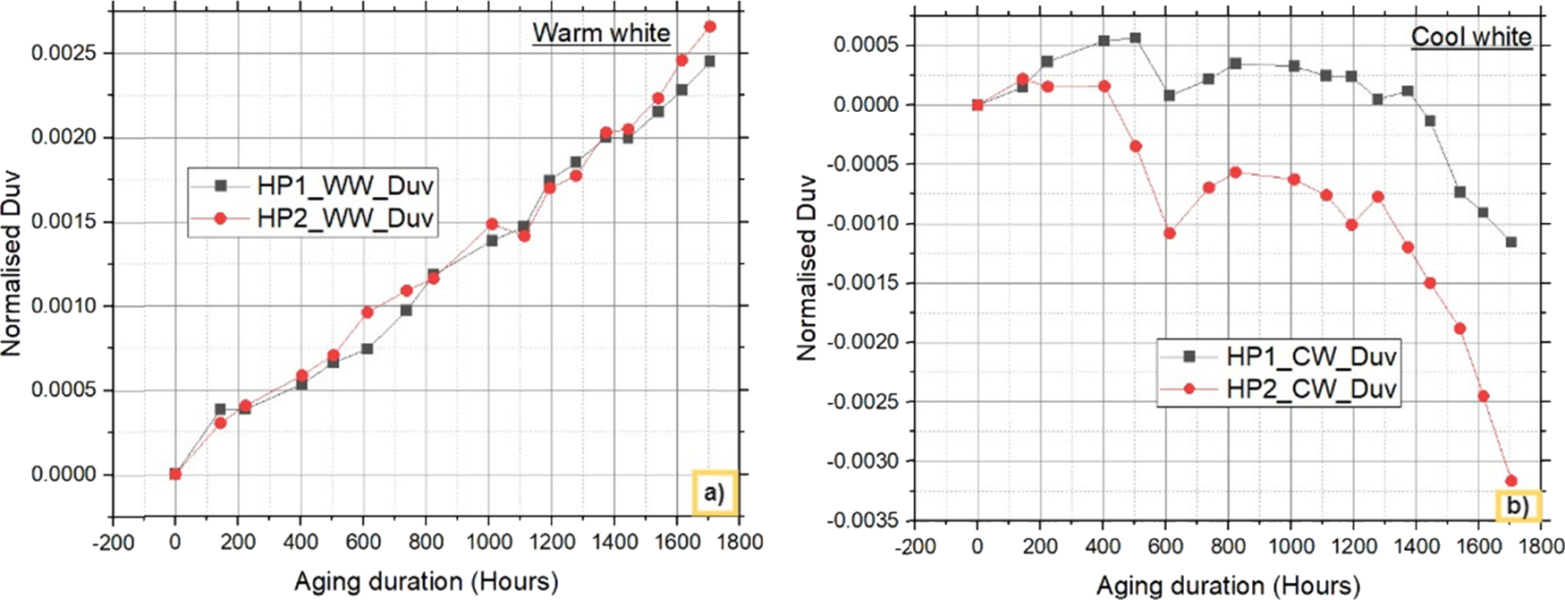
Variation in Duv with aging for a) warm white LEDs and b) cool white LEDs.

The colorimetric parameter Duv is analyzed to understand the contribution of phosphor. The variation in Duv over the study period is shown in Figure 12. The absolute differences in Duv between WW LEDs and CW LEDs are 0.00021 and 0.00200, respectively. This proves that the variation in the results between WW and CW LEDs is due to the phosphor, as the compositions of the phosphor in warm and cool white LEDs are different. The results were also verified by analyzing the blue and yellow spectra separately from those of the SPD and explained in an earlier section. The decrease in down converted light from CW LEDs is greater than that from WW LEDs.

**Figure 13:**
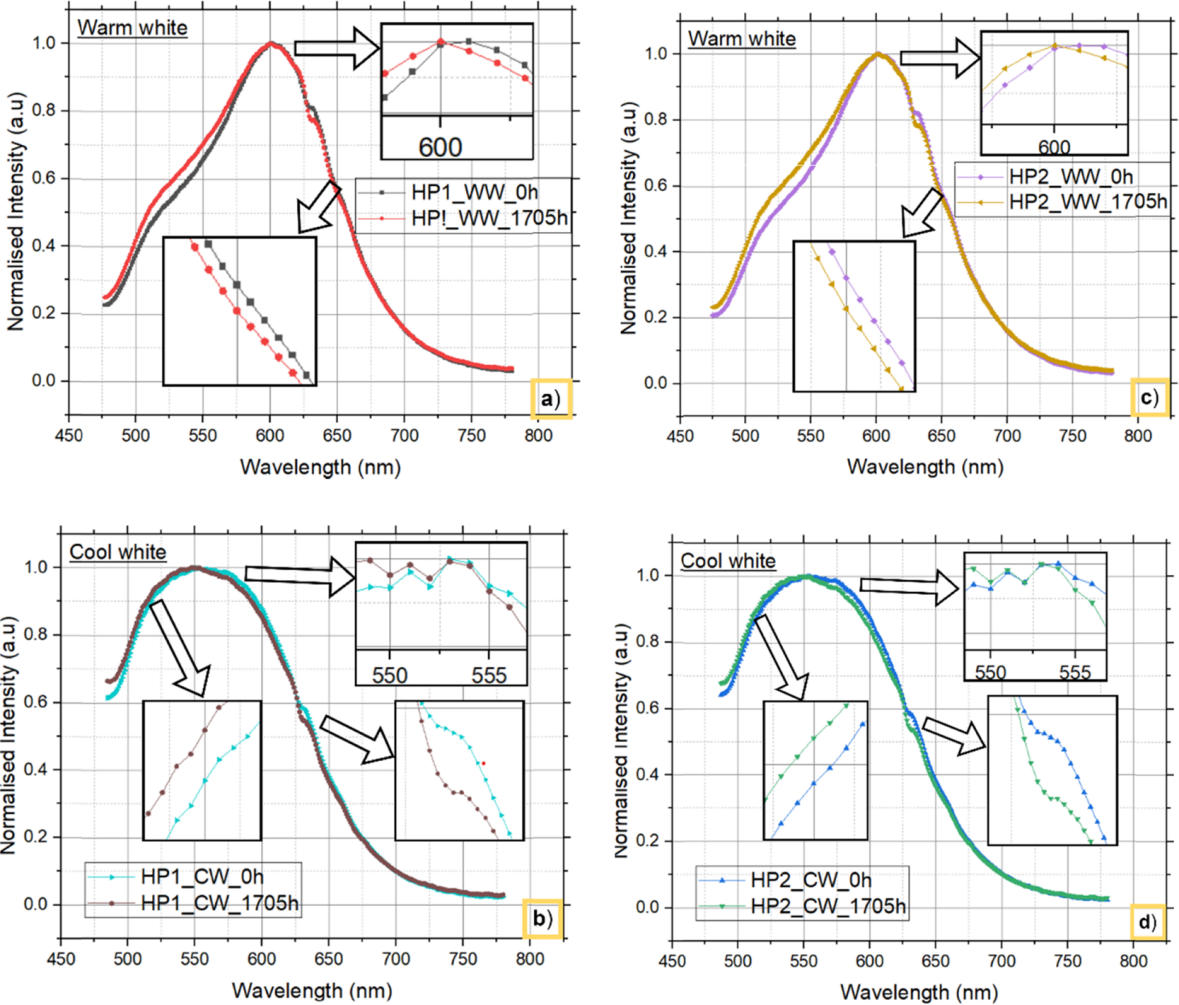
Normalized intensity plot of different LEDs: (a) HP1 (6 V)_WW, (b) HP1 (6 V)_CW, (c) HP2 (24 V)_WW and, (d) HP2 (24 V)_CW.

Furthermore, the phosphor layer was extracted from both zero-hour and degraded samples. Subsequently, photoluminescence studies were performed on these samples at an excitation wavelength of 450 nm. The intensity and wavelength spectra were collected for both the initial hours and the degraded LEDs of both LEDs. The intensity data were normalized with respect to the peak wavelength of the spectrum, and the findings are depicted in Figure 13.

In Figure 13, the normalized spectral intensity tends to increase between 470 and 600 nm, followed by a decrease above 600 nm in both configurations of WW LEDs. This shift is linked to changes in the lattice coordinate sites surrounding the emitter dopant, suggesting chemical transformations within the phosphor.^41^ Furthermore, the centroid wavelength using eq 1 was determined, revealing a decrease from 579.99 to 573.38 nm from HP1_WW, aligning with the anticipated shift in green chromaticity.^36^ Similarly, the centroid wavelengths for the other LED configurations are given in Table 5. The data indicate that the variance in the centroid wavelength between HP1_WW and HP2_WW is 1.29 nm, which is a smaller margin than the variance observed between HP1_CW and HP2_CW LEDs, which is 2.45 nm. This difference in centroid wavelength between the two CW LEDs contributed to the variation in the Duv results.

**Table 5:** Centroid Wavelength: HP1_WW & HP2_WW

	**warm white**		**cool white**	
**hours**	**HP1_WW**	**HP2_WW**	**HP1_CW**	**HP2_CW**
**0**	579.99	578.38	568.83	562.40
**1705**	573.38	573.06	565.05	561.08
**difference**	6.61	5.32	3.78	1.32

Upon analysis, it is clear that LEDs with different current and voltage configurations display variations in color despite having the same power. Additionally, the results also indicated a significant performance difference between different CCTs with the same power rating. Therefore, this study recommends conducting reliability studies on LEDs with various configurations, as they are available in the market. This approach will assist users in selecting the most suitable LEDs for their specific application requirements. Additionally, it is recommended that physics of failure analysis be performed on LEDs with different configurations. This approach helps in understanding the root causes of failures and contributes to obtaining better quality LEDs in the market.

## 4 Conclusion

A reliability study is conducted on commercially available LEDs of the same power, focusing on two main configuration categories: (i) different CCTs to analyze the phosphor contribution to LED failure and (ii) different voltage and current configurations. In addition to reliability analysis, photoluminescence, C–V, and I–V characteristic data are used to reveal the root causes of LED failure. High-power LEDs with a power rating of 3.84 W are used for the study. From the analysis, the following conclusions can be drawn: (i) The degradation rate between WW and CW LEDs varies. (ii) Cool white LEDs deteriorate faster than warm white LEDs do. (iii) The decrease in light output is primarily due to chip degradation, which is validated by electrical parameter analysis. (iv) Compared with WW LEDs, the 6 V, 640 mA (HP1_WW) configuration results in faster degradation than do the 24 V, 160 mA (HP2_WW) LEDs. Conversely, compared with the 6 V, 640 mA (HP1_CW) LEDs, the 24 V, 160 mA (HP2_CW) configuration results in faster degradation. (v) The variance in the percentage reduction in blue light between the HP1 and HP2 LEDs primarily arises from differences in lumen degradation between the WW and CW LEDs. This observation is further supported by comparisons of electrical parameters. vi) The contribution of the phosphor also plays a significant role in WW and CW LEDs results, as verified through Duv. The absolute difference in Duv between CW LEDs is greater than that between WW LEDs. On the basis of the above results, LED manufacturers should provide degradation analysis for each LED configuration available in the market. This information will assist luminaire manufacturers in selecting the right LEDs for luminaire design. Additionally, performing physics of failure analysis on each configuration of LEDs will aid in understanding the reasons behind LED failures, thereby contributing to the improvement of LED quality.
